# Is Robotics the real game changer for Urological cancer care during COVID-19 crisis?

**DOI:** 10.3126/nje.v11i2.38133

**Published:** 2021-06-30

**Authors:** Indraneel Banerjee, Indrajit Banerjee, Shantimoy Banerjee

**Affiliations:** 1 Consultant Uro oncologist and Robotic Surgeon, Apollo multi speciality Hospitals, Kolkata, West Bengal, India; 2 Sir Seewoosagur Ramgoolam Medical College, Belle Rive, Mauritius; 3 Senior Consultant, Orthopaedic Surgeon, Kalyani, West Bengal, India

## Background

The ongoing COVID-19 crisis due to the SARS-Cov-2 virus gained a pandemic status on March 11th, 2020 [1]. Although India did well during the first wave in the last year but the ongoing second wave and the triple mutated virus has devastated the whole nation and exposed the unpreparedness to fight this deadly disease. The death toll in the second wave has crossed 2 lakhs already [2]. We must also be aware that with the ongoing pandemic, care of another grave epidemic called cancer care has been severely affected. The reasons may be already overburdened health care system, fear of contracting the viral disease while visiting cancer care facilities, confusion surrounding the vaccination drive, and the travel restrictions due to the government enforced lockdown. Ranganathan et al. in their recently published cohort study in Lancet Oncology involving 41 hospitals in India have reported that, during the first wave last year about 70% of cancer patients in India were unable to undergo life saving surgeries and treatment and only 1/5th of the cancer surgeries was performed in March-May,2020 as compared to the same time frame in 2019. About 51,100 cancer surgeries were cancelled in India during the same time period [3]. It led to the cessation of cancer screening programs and increase in cancer stage migration, which will have a significant bearing in the cancer outcome in these patients in the future. In India, genitourinary cancers are the most common cancers in both sexes [4]. Genitourinary cancers in male comprises of cancers in the prostate 77.6%, urinary bladder, kidney, penis 11.6% and testis 10.5% [5]. Due to the COVID-19 outbreak the care of genitourinary cancer patients have also been compromised and several national and international bodies have come up with best practice guidelines to triage and treat urological cancer patients in a timely manner without putting undue stress upon already exhausted healthcare system [6-14]. Table 1 summarizes the triage protocol to be followed in Urological cancer patients for optimal cancer care.

### The dawn of Robotic surgery

Robot assisted laparoscopic surgeries (RAS) have revolutionized urological cancer care. The da Vinci surgical system (Intuitive Surgical Inc., Mountain View, CA, USA) was approved by US FDA on July 2000 and the first robotic surgery for Urological cancers in India was performed in All India Institute of Medical Sciences, New Delhi way back in July 2006 in the form of a Robotic Radical prostatectomy [15]. Since then, more than 85 da Vinci surgical systems have been installed in India till date (Intuitive unpublished data). With other surgical robots (Medtronic Hugo, SSI Mantra, Korean robot Revo etc) also being introduced in India and Health insurance companies coming up with plans that will cover these expensive surgeries, the future of robotics is looking great. There are certain advantages for the patients if they undergo robotic surgeries. There will be smaller incision and scars, less pain, minimum blood loss and patients can go home early. For the surgeons, the robots offer better 3 dimensional magnified (10-12x) HD vision, improved dexterity due to “endowrist” movements of the robotic arms and the robots can reach to the areas which are very difficult or even impossible to reach by conventional laparoscopy [16].

### Robotic surgery during COVID-19 pandemic

There are some major concerns surrounding minimally invasive surgeries that include laparoscopy and robotics during the pandemic. These surgeries involve abdominal insufflation with CO2, which increase intra-abdominal pressure and thus may increase the generation of aerosol leading to the risk of contamination with COVID-19 virus to the surgical team. The robotic surgeries can safely be performed with intra-abdominal pressure at 5mm of Hg using an intelligent integrated flow system (AirSeal ® system), as compared to traditional laparoscopy, which requires a pressure of 10-15 mm of Hg thus RAS can reduce contamination risk by reduced aerosol generation [17]. However, there is no proof that the aerosol released during minimally invasive surgery contains COVID-19 virus [9]. Apart from this advantage, robotic surgery needs fewer personnel in the operating room as compared to open surgery, lesser surgical instruments and thus faster cleaning and rapid turn over time. The surgeons’ console may be placed outside the operating room, thus safeguarding exposure to the surgeons. In most of the robotic urological cancer surgeries (except radical cystectomy for bladder cancer) same day or next day discharge is possible, which limits exposure for the patients and is relevant in the time of acute shortage of hospital beds [18]. Keeping these advantages in mind robot assisted surgeries can be a real game changer for managing complex urological cancer surgeries during the ongoing pandemic. Table 2 summarizes the precautions taken during robotic surgeries to prevent COVID-19 contamination.

### The future of Robotic surgery beyond the pandemic in India

Machine learning and AI platform from the data generated from the newer surgical robotic systems may pave in the way for autonomous robotic system in the future [19]. Research is ongoing to incorporate eye tracking, voice commands, tactile feedback and centralizing vital information to improve a surgeon’s experience [20-24]. The ultimate goal in a pandemic situation would be remote access no-contact robotic surgery under direct supervision of the surgeon. This may be a reality in the near future soon. The 5G internet service scheduled to be launched in India will permit real time signal transmission and thus allowing telesurgery in remote places [21]. With the launch of newer surgical robots, the initial purchase cost will come down and robotic surgeries will become more cost effective and tailor made for Indian patients.

## Conclusion

The mortality from COVID-19 infection is around 2-3%, but due to the delay in diagnosis and treatment the mortality from different cancers have increased significantly during the pandemic [25]. We know that the pandemic is going to stay a little longer, but the cancers will continue to kill even when the pandemic is over. Hence, to fight against cancer during this pandemic we should protect the health care workers, judiciously use telehealth, restrict the number of family members to accompany with the patient, use the resources wisely, have a well-planned outlook to treat different cancers and rely on newer technologies to tide over the crisis.

## Figures and Tables

**Table 1.** Simplified summary of the triage protocol to be followed in the management of Urological cancers during COVID-19

Site of Cancer	Cancer stage	Management
**Kidney**	cT1a	Postpone Sx for 6 months
	≥cT1b	Postpone Sx for 3 months
	Any T, Hematuria/ symptomatic/Renal vein/IVC involvement	Immediate Sx
	Metastatic RCC IMDC Good and Intermediate risk	TTX, CN after 3-6 month
	Metastatic RCC IMDC poor risk	TTX
**NMIBC**	Low risk	Postpone Sx for 3 months
	Intermediate risk	Prefer Sx
	High risk	Sx
	Any tumor with hematuria	Sx
**MIBC**	cT2N0	Trimodal therapy/Sx
	≥cT/ any N+	Sx within 3 months
	pT3/T4, p N1-N3	Defer adjuvant CT after Sx, Immuno preferred
	Metastatic bladder cancer	Defer CT, Immuno preferred
	Metastatic bladder cancer with hematuria	Hemostatic RT/Endoscopic fulguration
**Prostate**	Low risk	AS/Defer treatment for 6 months
	Intermediate risk	Defer treatment for 3-6 months
	High risk	Neoadjuvant ADT for 3-6 months followed by Sx/RT
	Metastatic	LHRH agonist preferred
	CRPC	Abiraterone/Enzalutamide preferred. Avoid Docetaxel CT.
**Penis**	cTis, cTa, cT1	Postpone Sx for 3 months
	cT2/cT3	Sx
	cT4	Sx + adjuvant CT
	**B/l negative groin**	
	Low risk	Surveillance
	Intermediate risk	Surveillance
	High risk	Sx postponed for 3 months
	Positive mobile nodes	Sx
	Positive fixed nodes/>4cm	Neoadjuvant CT followed by Sx
	Metastatic disease	Palliative CT
**Testis**	**Seminoma**	
	CS I Low risk	Surveillance
	CS I High risk	Surveillance/CT
	CS II A, IIB	CT/RT
	CS IIC, III	CT
	**Non Seminoma**	
	CS I A	Surveillance
	CS IB	Surveillance
	CS IS	CT
	CS IIA, IIB	CT
	CS II C, III	
	Good risk	CT
	Intermediate risk	CT
	High risk	CT

Sx: Surgery, TTx: Targeted therapy, CN: Cytoreductive nephrectomy, NMIBC: Non muscle invasive bladder cancer, MIBC: Muscle invasive balader cancer, CT: Chemotherapy, RT: Radiotherapy, AS: Active surveillance,

**Table 2.** Steps enumerating precautions to be taken during Robotic surgery in Urological Cancer patients to prevent contamination from COVID-19 infection

Workflow	Action to be taken
**1.Scheduling patients for surgery**	Postpone all non-emergency/non urgent procedures (vide table 1) Avoid surgery on COVID-19 positive patients (if applicable)
**2. Pre-operative office workup and screening of patients**	Prefer telehealth consultation Screening for Covid-19 symptoms, travel history and exposure history. Covid-19 RT PCR/HRCT chest in all patients posted for surgery. Counselling for possible risk of contracting Covid-19 infection during hospital admission.
**3.OR set up and Anesthesiology team**	>20 air changes/hour HEPA filters for air filtration. Cleaning of Robotic console head support between each case. Entry and exit in OR to be restricted Use PPE Use Video laryngoscopy for intubation HEPA filter attached to the endotracheal tube before intubation Minimize risk of aerosol formation Only the anesthesia team members should be present during intubation and extubating
**4.Robotic surgery team**	All cases are to be done by experts Surgeon console can be kept outside OR Only single bedside assistant. Bedside assistant should use PPE. Keep pneumoperitoneum at minimum (5 mmHg) and use Air Seal device. Minimize instrument entry and exit and minimize air leak Avoid ultrasonic sealing devices and keep the diathermy setting at minimum Use Air Seal to suck all the CO2 at the end of the procedure. If Air Seal device is not available use a smoke evacuator connected to a HEPA filter/underwater seal using sodium hypochlorite solution.

HRCT: High resolution CT, HEPA: High efficiency particulate air, OR: Operating room
